# Design and Testing of a Structural Monitoring System in an Almería-Type Tensioned Structure Greenhouse

**DOI:** 10.3390/s20010258

**Published:** 2020-01-01

**Authors:** Araceli Peña, Mercedes Peralta, Patricia Marín

**Affiliations:** 1 Research Centre CIAIMBITAL, University of Almería, Ctra. de Sacramento s/n, 04120 Almería, Spain; pmm213@ual.es; 2 Department of Computer Science, University of Almería, Ctra. de Sacramento s/n, 04120 Almería, Spain; mperalta@ual.es

**Keywords:** computer vision, structural monitoring, displacement, force, temperature, wind, tensioned wire, greenhouse

## Abstract

Greenhouse cultivation has gained a special importance in recent years and become the basis of the economy in south-eastern Spain. The structures used are light and, due to weather events, often collapse completely or partially, which has generated interest in the study of these unique buildings. This study presents a load and displacement monitoring system that was designed, and full scale tested, in an Almería-type greenhouse with a tensioned wire structure. The loads and displacements measured under real load conditions were recorded for multiple time periods. The traction force on the roof cables decreased up to 22% for a temperature increase of 30 °C, and the compression force decreased up to 16.1% on the columns or pillars for a temperature and wind speed increase of 25.8 °C and 1.9 m/s respectively. The results show that the structure is susceptible to daily temperature changes and, to a lesser extent, wind throughout the test. The monitoring system, which uses load cells to measure loads and machine vision techniques to measure displacements, is appropriate for use in different types of greenhouses.

## 1. Introduction

Greenhouse cultivation is widespread in the world and drives the economy in many regions. South-eastern Spain has the largest concentration of greenhouses with 30,000 ha, although according to 2018 data from the Chinese Ministry of Agriculture [1], China has the largest area in greenhouse cultivation at 3.7 × 10^6^ ha. Most greenhouses are plastic-covered structures, and their structural safety has received attention due to the high economic costs of losses from complete or partial greenhouse collapses caused by severe weather such as wind and snow [2,3,4]. The mechanical performance of different greenhouse structures has been studied by numerical methods [5] and finite elements for static loads [6] and dynamic loads [7]. For this type of structure, there are few real data that can be used to validate calculation models [8,9], due to the high economic cost of monitoring structural performance. Some types of greenhouses with inflexible cladding have been tested on a small scale in wind tunnels to obtain the pressure coefficients on multi-span duo-pitch roofs [10] or single-span roofs with different geometries [11]. Numerical calculations have primarily been experimentally verified with small models [12,13] and simple loads [14], which do not reflect the mechanical performance under complex loads [15]. Finite element calculation is a powerful tool, but the accuracy of the simulations of the generated models differs from reality due to the complexity of the joints between the elements of a structure and the loads that act on it [16,17]; therefore, tests that can support the calculations and improve their accuracy are needed.

The predominant type of greenhouse structure in south-eastern Spain is the Almería type, representing more than 94.3% of greenhouses [18]. These structures of these greenhouses consist of tensioned cables that rest on steel columns. Despite the high economic value of the facilities and the crops inside them, there are no regulations or structural calculations required, and the only safety guarantee is the experience of the builders.

The stability of structures that involve steel cables is limited by the effects of changes in air temperature, seasonal climate, or solar radiation, which can cause significant changes in the tensile loads acting on such cables; this aspect has been studied on cable-stayed bridges by various authors. For example, Cao et al. [19] analyzed part of the data collected during two years by means of a bridge health monitoring system (HMS) located in southern China. They used, among other things, GPS rovers for displacement or electromagnetic (EM) sensors for cable force, and determined that the estimated maximum temperature gradient was more than twice the original design value and slightly larger than the specification of different standards. From the monitoring (HMS) of bridges, increasingly widespread, it has been found that the variation of the tension on the cables is significant [20] and decreases by 3% when the ambient temperature increases by 10 °C, which affects other elements of the bridge, such as movement in the support pylons [21].

The image analysis technique, which can be affordably combined with optical devices and cameras for contactless and remote measuring that does not affect a structure, has been used in many areas to measure different phenomena in the fields of engineering, medicine and agriculture [22,23,24,25,26,27,28,29]. Video cameras and image processing techniques have been used recently to satisfactorily measure displacement in actual cable-stayed structures, such as bridges and buildings [30,31,32,33,34]. In addition, tensile strength of the cable installed on a bridge has been estimated by means of load cells [35]. The amount of data depends on the number of pixels per frame and the number of frames per second. The use of cameras to measure displacement in relation to temperature has also shown satisfactory results in structures for diaphragm walls or pipelines [36]. Non-contact radar sensors have been successfully and economically used in bridge monitoring, such as image by interferometric survey of structures (IBIS-S), especially in structural safety analysis. Allowing to relate the variation in the vibration frequencies thereof with the deterioration of materials and the appearance of damage to the girders [37], or to determine the displacement of the deck for both seaport and continental bridges [38], or to estimate the service life of bridges subject to deterioration or events such as truck impacts or earthquakes [39].

The accuracy of measurements is affected by the camera-to-target distance and the optical axis inclination, although these errors can be acceptable for small angles [29,40] and the accuracy starts to decrease at a distance of one metre or more [31]. However, camera placement is not critical [32] when known structural dimensions are used for calibration, but the camera axis needs to be perpendicular to the measured target. Regarding image analysis, work is being undertaken on the improvement, restoration, enhancement, and extraction of characteristics and on spatial and spectral texture analysis [33].

Structure modelling or calculation of Almería-type greenhouses is difficult due to the variability of joints between strength elements, the complexity of the grid of cables that make up the resistant structure, the method of load transfers, and the different natures of the materials, especially since there are no real data or a scale of structural behaviour with which to validate them. Monitoring structures under actual load conditions for a period of time allows their structural performance to be assessed and provides information on their state. This technique allows the development and adaptation of design and construction standards and the evaluation of structural safety under actual long-term load conditions. This article presents the monitoring design of one of these greenhouses, using load measurement devices for the structural elements (cables and columns) and a device to measure displacements in the columns using photographic cameras. The aim of this work is to understand the structural behaviour of this type of greenhouse and to have data with which to validate future calculation models by FE.

## 2. Description of the Recording Equipment and Analysis Techniques 

### 2.1. Greenhouse and Equipment for Environmental and Tensile Data Recording 

**Figure 1:**
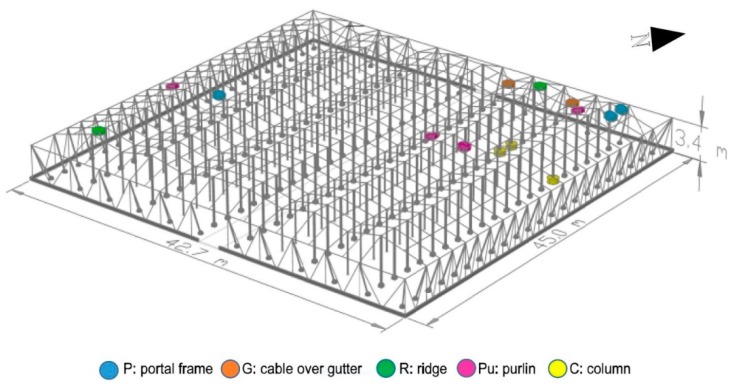
Perspective of monitored greenhouse with load cell locations.

The monitored greenhouse was an Almería-type greenhouse located in the practice field of the University of Almería (University of Almería—Anecoop Foundation’s Experimental Field Station). The NW–SE oriented and five span Almería-type greenhouse was made of a structure of wire braids tensioned on both the roof and the inside that only functioned under tensile stress, while the compressive stresses were borne by the inner columns (Figure 1). These columns were hollow galvanized steel tubes with a diameter of 9 cm, a thickness of 2 mm, and a height of 4.7 m. It was a complex system at equilibrium for which all joints were hinged. 

**Figure 2:**
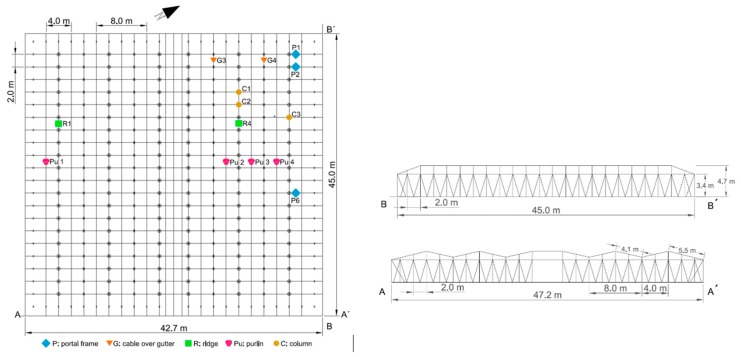
Greenhouse where the tests were conducted, dimensions (in m) and load cell locations.

The plastic greenhouse consisted of five spans with a gable roof (Figure 2), with span width 8 m, the height at the cable over gutter was 3.4 m and the ridge 4.7 m. The roof was a double meshing of longitudinal cables over the highest and lowest points and the centre of both points (purlins); these cables rested over transversal cables (portal frame) separated at a distance of 2 m. The plastic sheet enclosure was located between the two wire grids [18]. 

**Figure 3:**
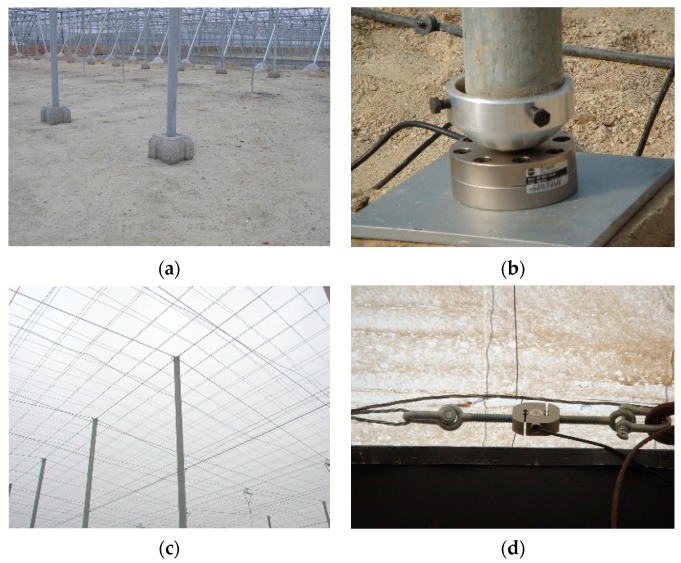
(**a**) Column without load cell. (**b**) Column with compression load cell installed. (**c**) Greenhouse roof. (**d**) Load cell on roof cable.

**Figure 4:**
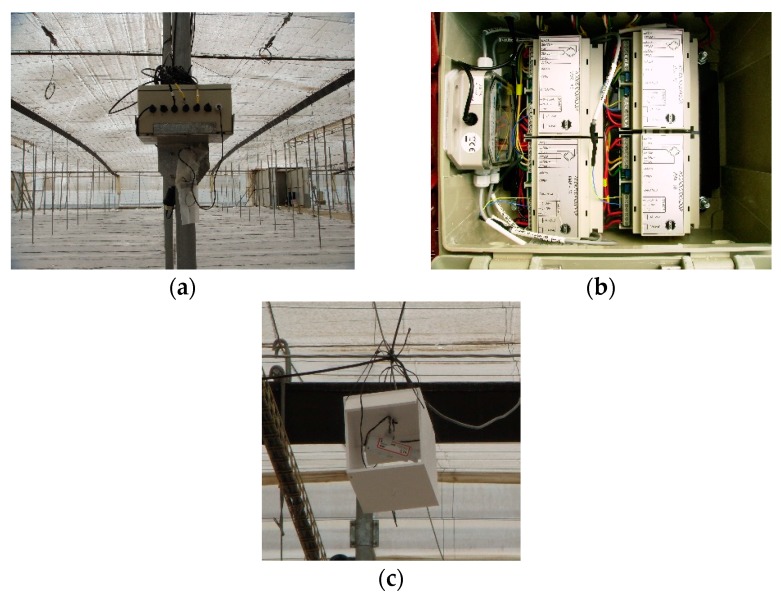
(**a**) Recording device box. (**b**) Box interior. (**c**) Greenhouse indoor temperature sensor.

The columns rested directly on a concrete piece (Figure 3a) that was placed without being attached to the ground. To monitor the transferred load, three compression load cells capable of measuring up to 70,000 N (Figure 3b) were placed at the base of the column (Figure 1). The roof cables were made up of three threads (Figure 3c) of braided wire supported longitudinally and transversely on the head of the column (Figure 3c). The tensile forces were measured by 11 load cells, each of which had a capacity of up to 50,000 N, that were placed (Figure 1) in the cables (Figure 3d). The recording devices, load cell conditioner and Hobo station were placed in airtight boxes to avoid moisture (Figure 4a,b). Each Hobo station had four inputs. Temperature sensors were installed close to the load cells to determine the effect of temperature on stress (Figure 4c). 

The location of the load cells in the different resistant elements of the greenhouse was carried out considering the following criteria: the greatest distance free of obstacles that interfere with the action of the wind on the structure corresponded to the north face with about 30 m, while in the other three the distance between greenhouses of similar height to the one tested was only 4 m; the greatest intensity and direction of the winds in the area corresponded to N, NNW, and NNE [41]. According to these criteria, we expected that the northwest corner of the greenhouse would be the most affected by the wind, so we had a higher number of load cells in the resistant elements of the greenhouse in this area.

The temperature, wind direction, and speed outdoor were measured with a nearby meteorological station, placed where the interaction from the structure was negligible, at a height of 10 m equipped with a Pt1000 temperature sensor and a cup anemometer measurement range 0 to 40 m s^−1^ (accuracy ± 5%). Wind direction was measured with a vane (accuracy ± 5°). In structures with a high sensitivity to wind, it is necessary to know both its direction and speed [42]. The suction load due to wind in windward and leeward roofs is greater when the wind is oblique, producing an increase in the axial force on the columns, which is more considerable if case of supporting columns [43].

### 2.2. Data Recording Equipment Implementation and Analysis of the Process of Machine Vision Control to Capture Displacements 

**Figure 5:**
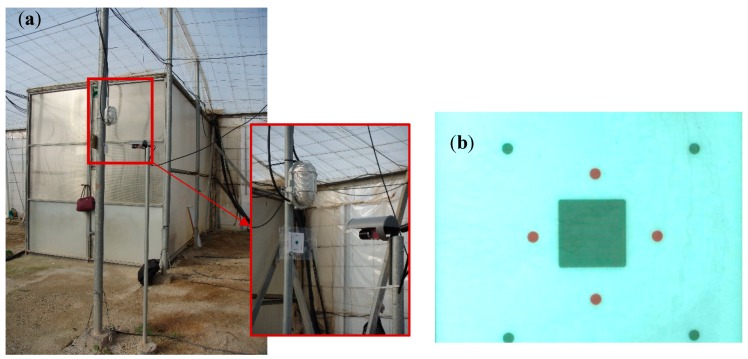
(**a**) Image capturing equipment. (**b**) Pattern image.

The method of measuring displacements in the columns using machine vision that was developed in this study consists of equipment located inside the greenhouse to record and store data that are then processed in the laboratory (Figure 5a). Image capturing was done in real time using two types of charge coupled device (CCD) cameras that collect images at a rate of 1 image/s, with resolutions of 584 × 480 and 640 × 480 pixels per image, and the capture oscillations in the structure had a frequency equal or less than 0.5 Hz.

Displacement measurement was performed on a pattern of visual elements with different tones under different lighting conditions and from which the pattern image was obtained (Figure 5b). This device was oriented in the plane perpendicular to the focal axis of the camera at a distance of 20 cm and attached to the column. Subsequently, image processing was carried out using a computer with an Intel Core^®^ Quad-core processor and a system consisting of three CUDA graphics processing units (GPUs) with 240 processing cores each (720 total processing cores). The resolution ranged between 9 and 12 pixels per millimetre depending on the camera used.

### 2.3. Image Processing

**Figure 6:**
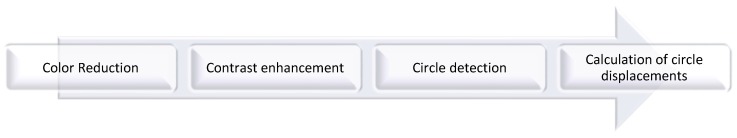
Diagram of image processing for displacement measurements.

Our study required techniques for colour conversion to greyscale, image improvement and enhancement, and the location of geometric elements. Figure 6 shows the image processing steps.

The system, which is summarized in Figure 6, generated a sequence of several thousand images each day that were downloaded periodically for processing. 

**Figure 7:**
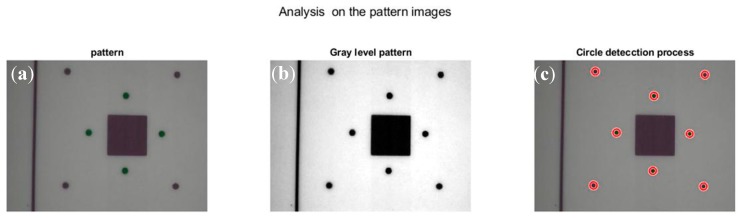
(**a**) Pattern image. (**b**) Grayscale image. (**c**) Image with circle detection.

First, colour reduction was performed to convert the image to black and white and to enhance the contrast (Figure 7a). 

This study used images in RGB format, which provide information in three matrices based on the colours red, green, and blue. The colour removal process was a transformation of $C^{3}\overset{f}{\Rightarrow }T$, where C and T are the domains of representation in the colour space and the greyscale space, respectively. This process achieved a greyscale image with the same contrast, sharpness, shade, and structure of the colour image, as explained in [44]. 

The conversion of a colour image to greyscale in real time, was performed using an algorithm [45] that enables sampling and reduced dimensionality through Gaussian pairing techniques and colour difference analysis on major components. Based on an application of the work developed by [45], the process of transforming an image encoded in the RGB colour space could be performed using an expression that allows a greyscale image to be obtained from the expression

T=0.333∗R+0.5∗G+0.1666∗B

where *R* is the red component of the image, *G* is the green component and *B* is the blue component. This is the transformation used in MATLAB to convert RGB images into greyscale images, which is effective in the analysis of pattern images.

Subsequently, the image needed a tone adjustment, which improved the image contrast by revealing the existence of significant elements (Figure 7b). Tone adjustment processes were performed using the histogram of tone frequencies of the image in grey. This adjustment was made by converting the image histogram into a plane histogram with similar frequencies in all the image tones [46], which improved the sharpness and contrast. This study used a process of expanding the histogram of the image tone frequencies from 10% to 90% of the range (from 0 to 256), using a linear transformation:

$$T:\left[i,s\right]\overset{f}{\to }\left[0.1*li,0.9*ls\right]$$

where *i* and *s* are the lower and upper tone limits of the image, respectively, and *li* and *ls* are the lower and upper limits, respectively, of the maximum tone that any image can take. The simplest transformation is a linear transformation.

T(x)=(x−i)∗(0.9*ls)/(s−i)

The detection of circles in the measurement patterns on the image (Figure 7b) was then carried out applying the methodology based on the technique of image analysis using the Hough transform, which was developed by several authors [47,48,49]. This technique involved passing the image elements to a parametric space, from which circumferences, straight lines, and other simple geometric shapes are reconstructed (Figure 7c).

**Figure 8:**
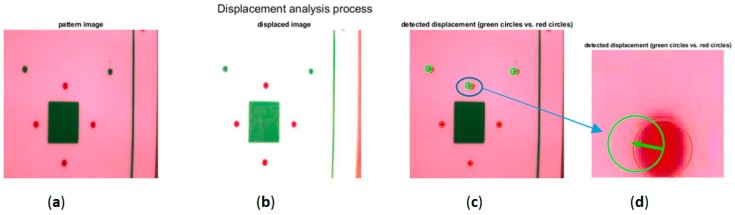
(**a**) Pattern image (**b**) Studied images (**c**) Displacement detection (**d**) enlargement of a detail (**c**).

Lastly, the displacement was measured by detecting whether the circles of an image (Figure 8b) had moved (Figure 8c) in relation to the pattern image circles (Figure 8a). To do this, the positions of the centres of the circles of each image were detected and were compared to those of the pattern image; red circles represent the positions of the marks in the pattern, and green circles represent the positions of the marks in the studied image (Figure 8c). The displacement and its angle were obtained immediately by calculating the Euclidean distance between the two centres. The length of the arrow (Figure 8d) is the linear displacement projected in the plane of the camera, and the measured angle between this vector and the horizontal was used to determine the direction and sense of the movement.

### 2.4. Measurement System Calibration

**Figure 9:**
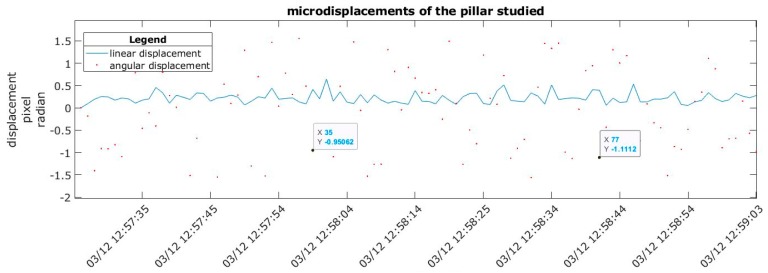
Displacements recorded between 03/12/2018—12:57:25 and 03/12/2018—12:59:03 at a resolution of 12 pixels/mm.

By analysing the micro-displacements that occur at frequencies of 0.5 Hz or less for short time intervals, the sensitivity of the measurement system was determined. For one image, displacements were random and meaningless. Likewise, the orientation of these displacements was not significant, and the angles (red points) were distributed uniformly (Figure 9). This indicates the natural behaviour of the column, regardless of environmental factors. The maximum linear displacement was 0.04 mm, and the maximum angular displacement 1.5 radians.

## 3. Results and Discussion

The displacements obtained in three periods (Table 1) were analysed to obtain the displacements and loads on the measured strength elements, depending on the environmental variables of wind speed, wind direction and temperature.

### 3.1. Displacement Results

Displacements throughout one day were calculated using 1 image/2 min, and it was observed that the structure oscillated between a maximum and a minimum. In general, all linear displacements were small at 0.25 mm, 0.63 mm, and 1.13 mm in periods 1, 2, and 3, respectively.

**Figure 10:**
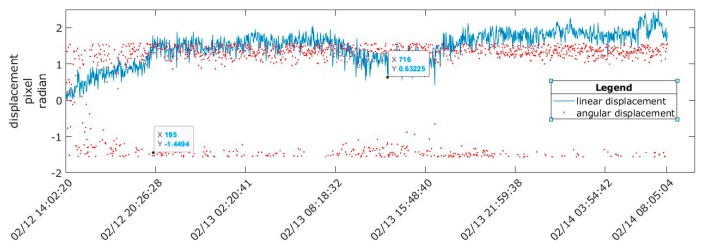
Linear and angular displacements for period 1.

**Figure 11:**
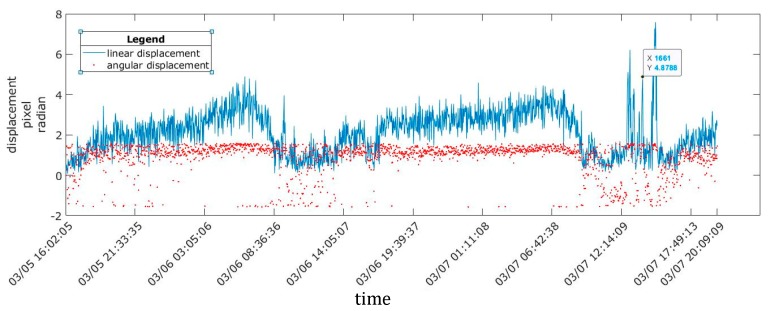
Linear and angular displacements for period 2.

**Figure 12:**
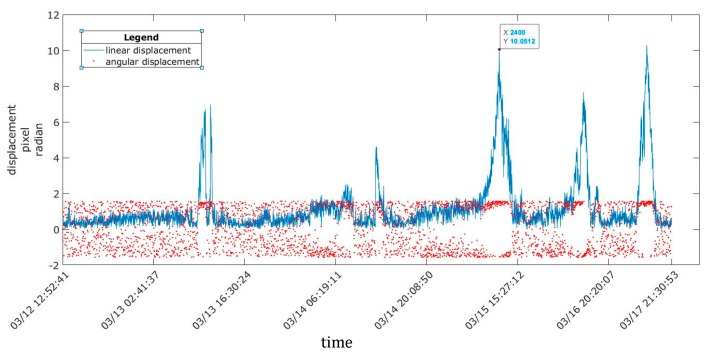
Linear and angular displacements for period 3, with five significant displacement intervals.

In all three periods, the maximum displacement values were obtained in the middle of the day (12:15–13:30), and the minimum values were obtained at approximately 6:00 in the morning, coinciding with the maximum and minimum temperatures inside the greenhouse. At the same time, the angular displacements were concentrated in the upper area of the space, which indicates that the direction and sense of the displacement were related to the wind direction (Figure 10, Figure 11 and Figure 12).

The displacement ranged between 0.16 mm and 0.38 mm in period 2 and was steady for several minutes, with a maximum of 0.63 mm (Figure 11). During period 3, (Figure 12), there were five significant intervals of displacement at 0.77 mm, 0.51 mm, 1.12 mm, 0.85 mm, and 1.13 mm, which corresponded to the period of maximum temperature rise between the morning and noon; hereafter, these subperiods are denoted as 3a, 3b, 3c, 3d, and 3e.

### 3.2. Results of Load Measurements in the Structural Elements 

Because very small displacements were registered in period 1, periods 2 and 3 were used to analyse the variation of the axial loads in the roof cables of the greenhouse and in the columns.

#### 3.2.1. Roof Cable Performance

**Figure 13:**
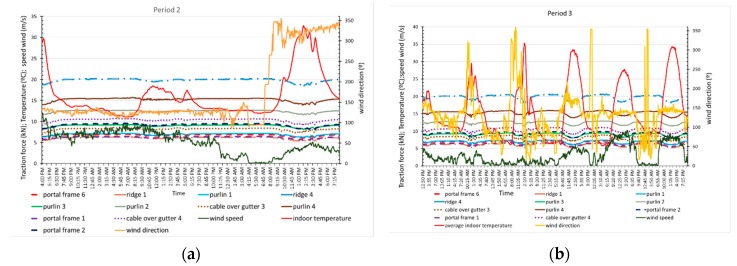
Traction force variation in the roof cables due to environmental variables in period 2 (**a**) and period 3 (**b**).

The cables of the greenhouse roof transmit the load through their support on the columns, and the load must be correlated with the load on the columns. The axial tensile load acting on each of the cables monitored is analysed below in relation to the environmental variables (Figure 13a,b).

Figure 13 shows that the position of the cables over the ridge is not indicative of a higher or lower load recorded. For example, the three cables located at the ridge (backbone) had recorded values that were very different from each other, with one under the highest tension and the other under the lowest. This may be due to the way that the greenhouse was built, with different initial tensions applied to each cable before the columns were installed depending on the judgement of the builder and not measured during construction. This result may also be due to cable loosening caused by the joint sliding and tension redistribution that occurs in this type of structure throughout its life. It was found that the elements located further to the northwest registered higher tensile loads, regardless of the wind speed, which can be significant, with values up of to 9 m/s. In all roof cables, the maximum tensile load occurred early in the morning, and the minimum occurred in the middle of the day. These values coincided with the minimum and maximum temperatures inside the greenhouse and produced load variations from 22% to 11.5% in each cable and temperature variations of 30 °C. The maximum wind values occurred in the middle of the day, but did not coincide with temperature maximums; the tensile loads fluctuate from 11% to 15% for speeds of 10.2 m/s and a temperature increase of 18 °C. The results are consistent with those obtained by other authors who studied bridges, in which the load on the bridge cables increased when the daily temperature was at a minimum and was lowest at the maximum temperature, [50]; 10% variations in the cable load were found when the temperature varied by 30 °C [51]. Cable load variations throughout the day are important and should be considered in future structural calculations of greenhouses, as other authors have proposed for bridge structures [52]. 

#### 3.2.2. Column Performance

**Figure 14:**
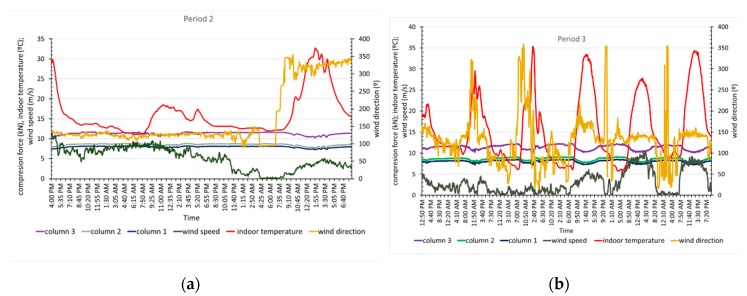
Compressive load variation in the columns with environmental variables in periods 2 (**a**) and 3 (**b**).

Due to the method used to build this type of greenhouse, the loads on the columns vary with their position, and in addition, the columns are only under compressive stress. The load was measured in three columns (Figure 1). Columns 1 and 2 were consecutive and spaced 2 m apart, with similar loads recorded. Column 3 was located farther to the northwest, and at 8 m in the transverse direction, it registered the highest compressive load. The structural performance of the columns in relation to the environment variables of temperature inside the greenhouse, wind speed, and direction are discussed below (Figure 14a,b).

The maximum compressive loads recorded in the three columns occurred between 6:00 and 7:30, which coincide with the lower temperature values inside the greenhouse; meanwhile, the minimum values were reached between 12:00 and 14:30, when the temperatures were at a maximum. The load variation recorded in these periods is between 13% and 17%. When the wind speed was below 6 m/s, the maximum and minimum load values in columns 1 and 2 were measured with 5-min to 20-min differences compared to column 3, which is the column closer to the windows. As seen with the roof cables, the maximum wind speed did not coincide with the extreme load values.

**Figure 15:**
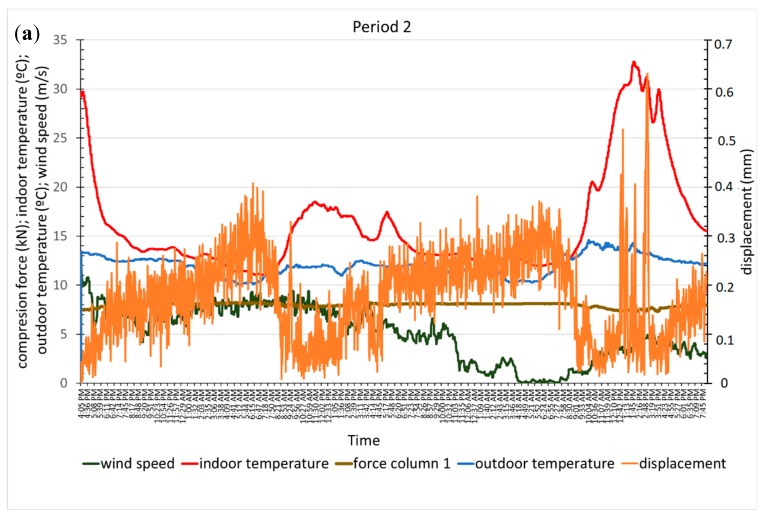

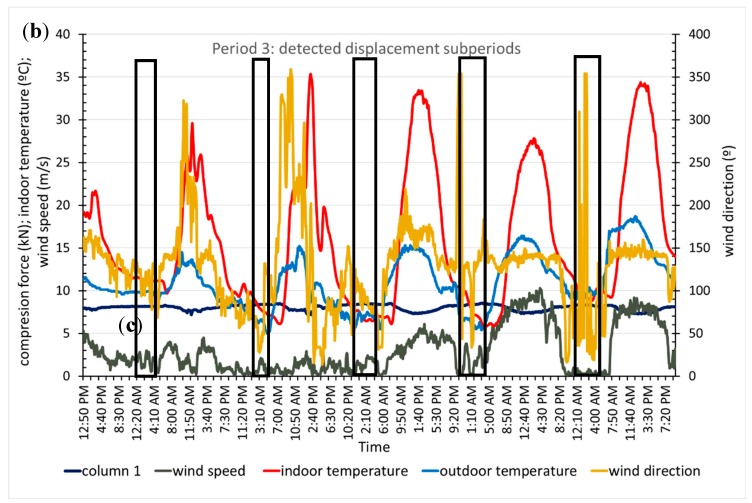
Graph of column displacement and environmental variables, period 2 (**a**) and subperiods of period 3 in which we detect displacement 3 (**b**).

Significant displacements in column 1 throughout period 2 and during five-time intervals in period 3 (Figure 15b) were recorded with the image analysis technique, with the maximum values occurring in the early hours of the morning and at noon. The displacement curve in period 2 (Figure 15a) showed a behaviour opposite to that of the temperature and more similar to that of the compressive load recorded in the column, with smaller displacement values seen at higher indoor temperatures and lower compressive loads. At the end of this period, two displacement maximums that occurred with northwest winds coincided with the higher temperatures, lower wind speeds and lower column loads observed when the winds were from the southeast. This displacement increase in the column could be associated with the wind direction and gusts that blow in the same direction, which were not recorded [53].

**Figure 16:**
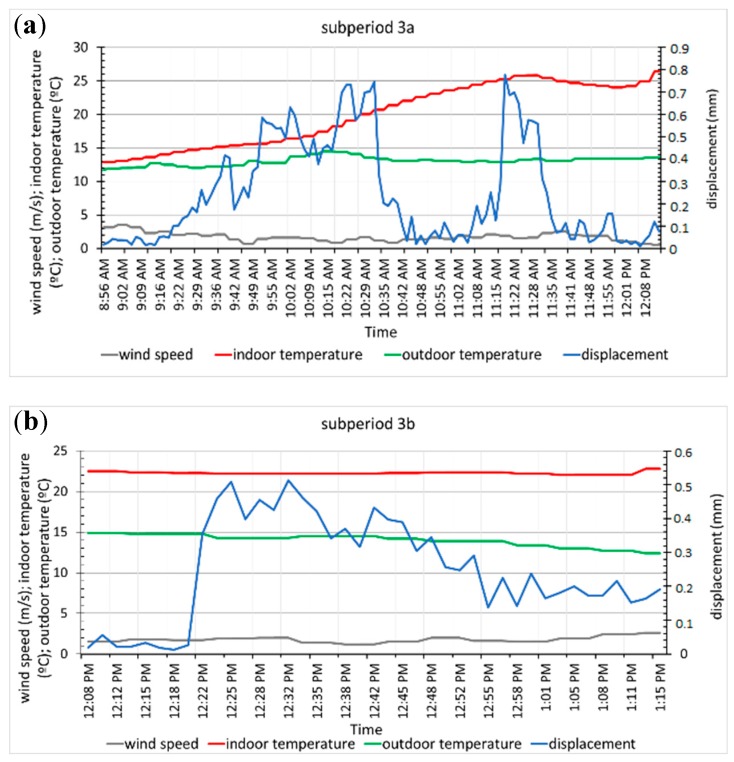

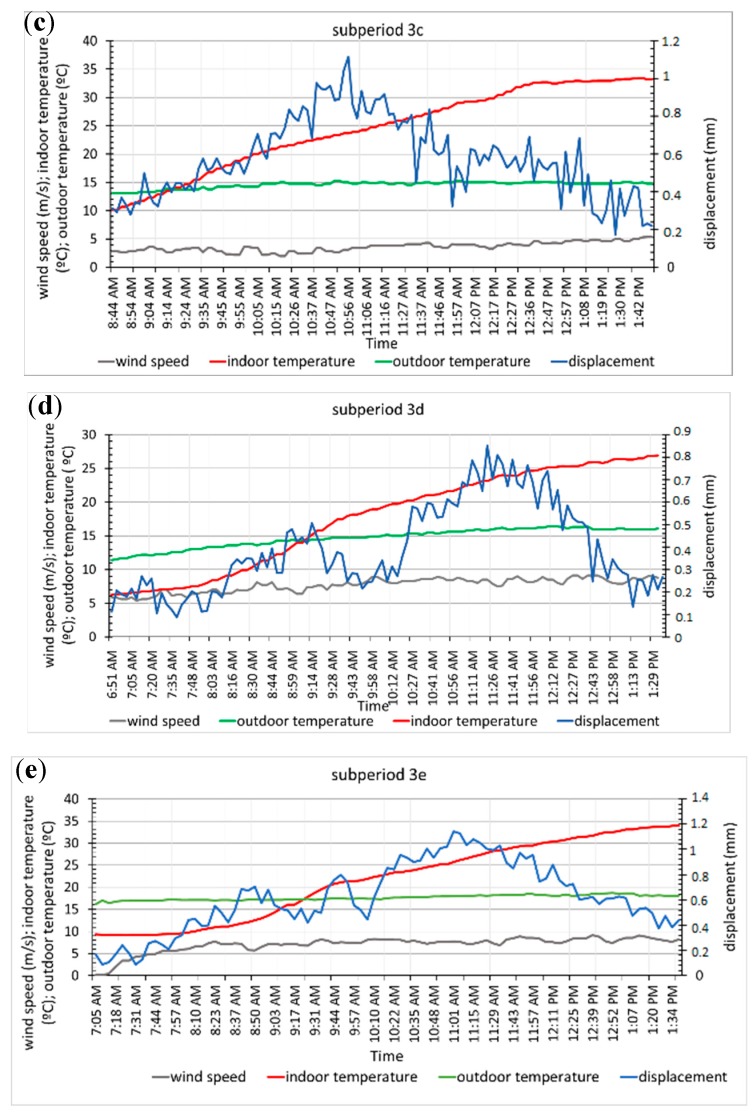
Column displacement and environmental variables in subperiods 3a, 3b, 3c, 3d and 3e of period 3 (**a**–**e**).

In general, for wind speeds below 5 m/s, the wind direction appears to influence the displacement because the displacement was smaller for NW winds (Figure 15a and Figure 16b) than for SE winds (Figure 15a and Figure 16a,c–e). Maximum speeds between 6 m/s and 9.2 m/s were recorded for SE–SSE winds in all cases, and the largest displacements do not coincide with either the maximum wind speed or the maximum temperature inside the greenhouse.

During the recording of the displacements, the temperature outside the greenhouse had small variations, while it increased continuously inside the greenhouse. The relative and absolute displacement maximums in both periods (Figure 15 and Figure 16) occurred when the outdoor temperature dropped between 0.2–0.5 °C and the wind speed increased by 0.1–0.7 m/s. The relative and absolute minimums occurred when the outdoor temperature dropped or increased between 0.1–0.4 °C, while the wind speed increased or decreased between 0.4 and 0.7 m/s.

The records obtained in the two periods show that the displacement depends on the axial compressive load on the column, which in turn depends on the indoor temperature (Ti) and wind speed. A multivariate linear regression analysis using Statgraphics Centurion XVIII software (Table 2) was carried out to determine the degree of correlation between all these variables. Table 2 shows statistical parameters, with R-square statistic indicates in which % the adjusted model explains the variability, indicating a relatively strong relationship between the variables, *p*-value ≤ 0.05 indicates that the variables considered have a statistically significant relationship, the Pearson’s moment (Coeff.Corr.), between each pair of variables, which varies from −1 to +1, and measures the strength of the linear relationship between variables. No significant statistical relationships (*p*-value < 0.05) were found between the displacement, the temperature inside the greenhouse and the wind speed. However, the column displacement was found to be statistically correlated in period 2, subperiod 3d, and subperiod 3e during which the wind speeds were higher, and the column compressive loads were lower compared to the other periods. Wind has a statistically significant relationship with the load recorded in the column, except in periods 2 and 3d during which the temperature was lower. In all cases, the compressive load is strongly correlated with the temperature inside the greenhouse.

**Figure 17:**
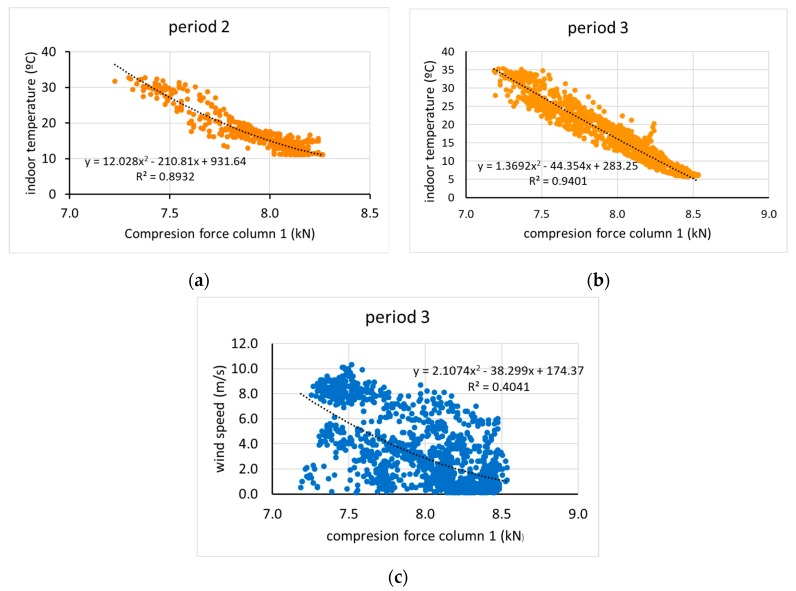
Relationship between force column and indoor temperature in periods 2 (**a**) and 3 (**b**), and between force column and wind speed in period 3 (**c**).

The failure to find statistically significant relationships does not mean these do not exist, especially when these seem to appear in the log curves. The regression fit found between the compressive load of the column and the temperature in the two periods was good (Figure 17a,b), and it was low between load and wind speed in period 3 (Figure 17c). 

**Figure 18:**
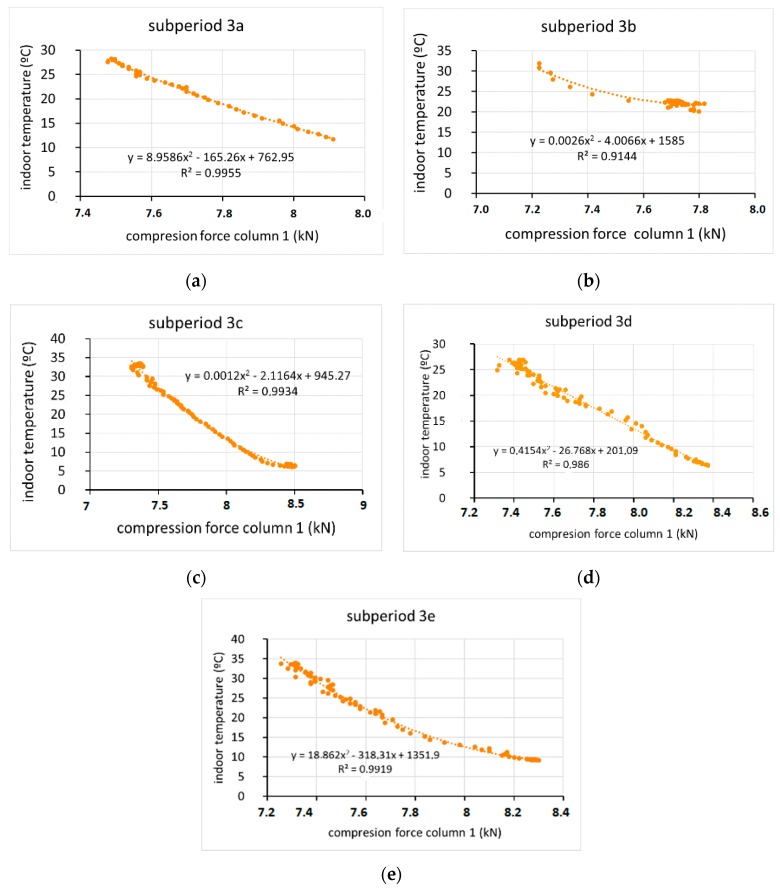
Relationship between compression force column 1-indoor temperatures in 5 subperiods (3a, 3b, 3c, 3d and 3e) of period 3 (**a**–**e**).

**Figure 19:**
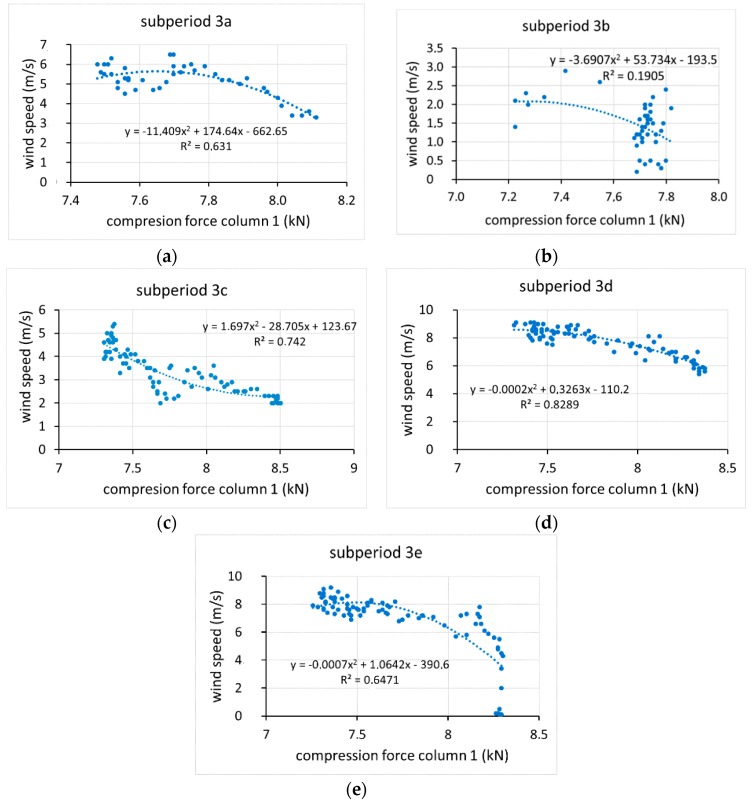
Relationship between compression force column 1-wind speeds in five subperiods (3a, 3b, 3c, 3d and 3e) of period 3 (**a**–**e**).

Because a relationship between wind speed and the compressive force on the column was found only in period 3, the intervals in which movement was recorded in that period (Figure 17) are analysed below. It was found that the load on the column was highly correlated with the temperature of the column, with R values greater than 0.9 (Figure 18). However, the fit with wind speed was less (Figure 19), and the interval with the best fit was period 3d, with R = 0.83 (Figure 19d). In general, wind speed influenced the tension of the column, although its influence was greater at lower temperatures. In period 3e, the compressive force was closely related with temperature (R = 0.992) (Figure 18e) but not with wind (R = 0.65) (Figure 19e). It follows from these results that the effects on displacement are greater with temperature variations than with wind speed variations. These results agree quite closely with those found for the monitoring of a bridge in Scandinavia [21], for which steel temperature changes dominated the measured displacement as a result of the thermal expansion caused by ambient temperature changes, although in that case, a 14.5 m/s wind speed had no effects.

## 4. Conclusions

Machine vision measurement technique can be effective for recording both linear and angular displacements and provide information about events related to the structural dynamics of greenhouses that are independent from wind effects.

The load on the strength element of the greenhouse is correlated with the ambient temperature of the greenhouse. It decreases when the temperature increases and increases when the temperature decreases according to daily temperature cycles.

The variation of the compressive loads on the inner columns is less than that on the lateral columns due to the lower temperatures caused by the entry of cold air, which increases with wind speed.

In general, column displacements increase when the compressive load on the column decreases, which occurs when the temperature inside the greenhouse is higher. Maximum displacements occur with small decreases in the outside temperature, while the inside temperature increases.

Column displacement is affected by the speed and direction of the wind and the temperature inside the greenhouse. The effect of wind speed increases when the temperature is lower.

These results show that the vulnerability of greenhouse structures is greater during the day when temperatures rise considerably, and in general, this period also coincides with higher wind speeds.

## Figures and Tables

**Table 1:** Test periods.

Period	Start Date	Start Time	End Date	End Time	Total Hours	Camera Resolution
1	02/12/2018	14:02:20	2/14/2018	8:05:04	42 h	12 pixels/mm
2	03/05/2018	16:02:05	03/07/2018	20:09:09	52 h	12 pixels/mm
3	03/12/2018	12:52:41	03/17/2018	21:30:53	128 h	9 pixels/mm

**Table 2:** Summary of key statistical parameters.

Period	Column Force-Ti			Column Force-Wind			Column Force-Displacement		
	R-Squared	Coeff. Corr.	*p*-Value	R-Squared	Coeff. Corr.	*p*-Value	R-Squared	Coeff. Corr.	*p*-Value
2	87.91%	−0.937	0.000	0.017%	0.013	0.57	22.01%	0.469	0.0000
3a	88.96%	−0.943	0.0000	19.26%	0.439	0.0000	0.605%	−0.077	0.4002
3b	16.55%	−0.407	0.0083	34.11%	−0.584	0.0001	0.033%	−0.018	0.9099
3c	97.26%	−0.986	0.0000	64.51%	−0.803	0.0000	0.508%	0.0713	0.4388
3d	87.15%	−0.933	0.0000	9.12%	−0.302	0.058	87.08%	0.933	0.0000
3e	95.55%	−0.977	0.0000	57.66%	−0.759	0.0000	43.35%	−0.658	0.0000
